# Correction: *Yersinia pestis* Activates Both IL-1β and IL-1 Receptor Antagonist to Modulate Lung Inflammation during Pneumonic Plague

**DOI:** 10.1371/journal.ppat.1004921

**Published:** 2015-05-13

**Authors:** The PLOS Pathogens Staff

## Notice of Republication

This article was republished on April 27, 2015, to insert information omitted by the publisher, specifically an additional author, Figure S7, and some text in the Results section. The publisher apologizes for the errors. Please download this article again to view the correct version. The originally published, uncorrected article and the republished, corrected article are provided here for reference.

## Supporting Information

S1 File Originally published, uncorrected article. (PDF)

S2 File Republished, corrected article. (PDF)
