# Supplementary figures and images for: Yersinia pestis Activates Both IL-1β and IL-1 Receptor Antagonist to Modulate Lung Inflammation during Pneumonic Plague
Source: PLoS Pathog. 2015 Mar 17;11(3):e1004688. doi: 10.1371/journal.ppat.1004688 (PMC4363893; doi:10.1371/journal.ppat.1004688)

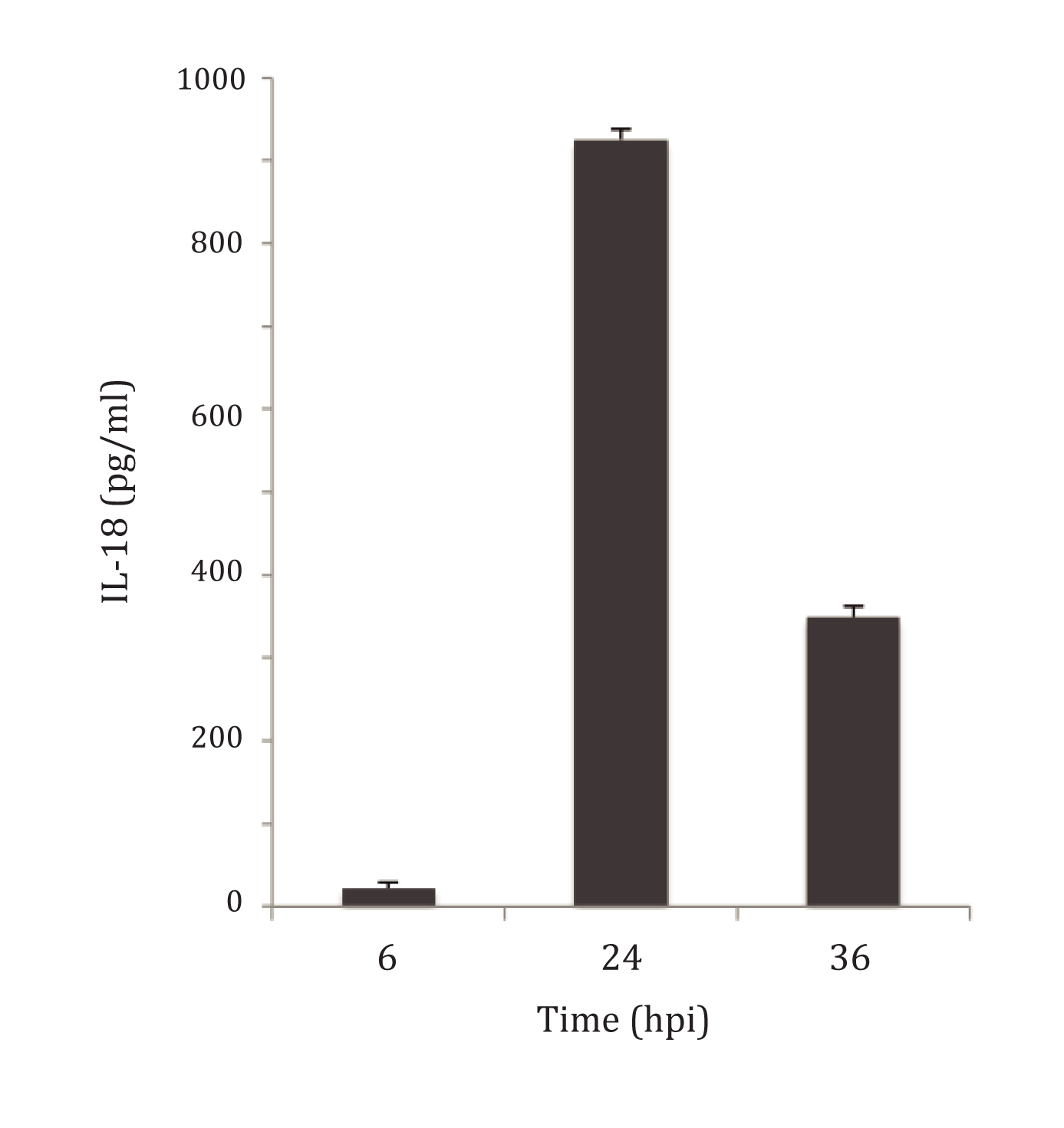

Supplement: S1 Fig — Mice were intranasally inoculated with fully virulent Y. pestis stain CO92, and sacrificed at 6, 24 and 36 hpi. Lung lysates were assessed for IL-18 cytokine protein using ELISA. N = 3 per time point. (TIF) [file ppat.1004688.s001.tif]

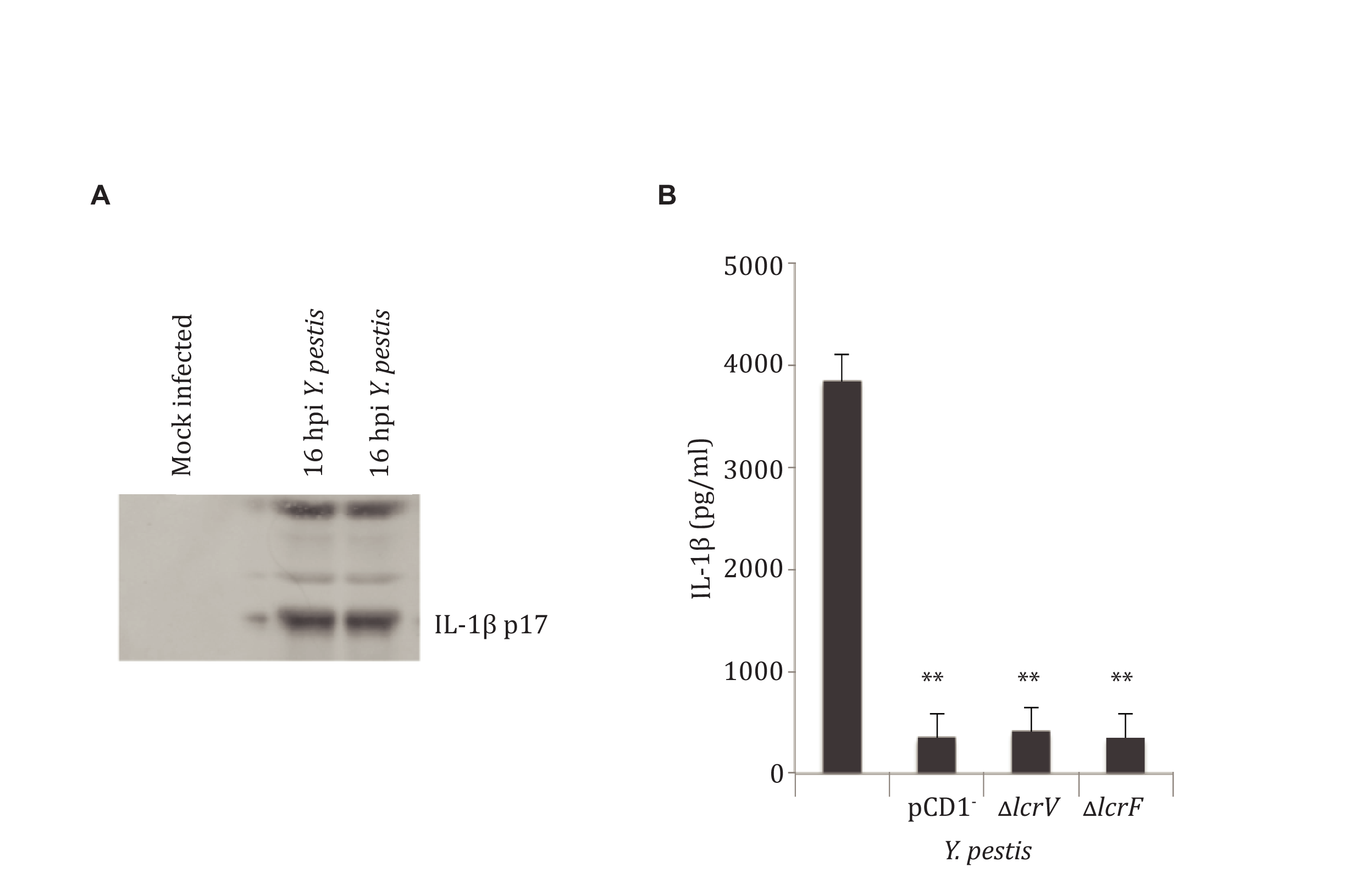

Supplement: S2 Fig — A. IL-1β Western blot analysis performed on supernatants obtained from bone marrow-derived macrophages (BMDMs) infected with Y. pestis at MOI 10. B. IL-1β ELISA performed on supernatants obtained from BMDMs 24 hpi with Y. pestis strains CO92, CO92 pCD1-, CO92 ΔlcrV, and CO92 ΔlcrF, at MOI 10. All in vitro infections were performed in triplicate, with representative analysis shown. (TIF) [file ppat.1004688.s002.tif]

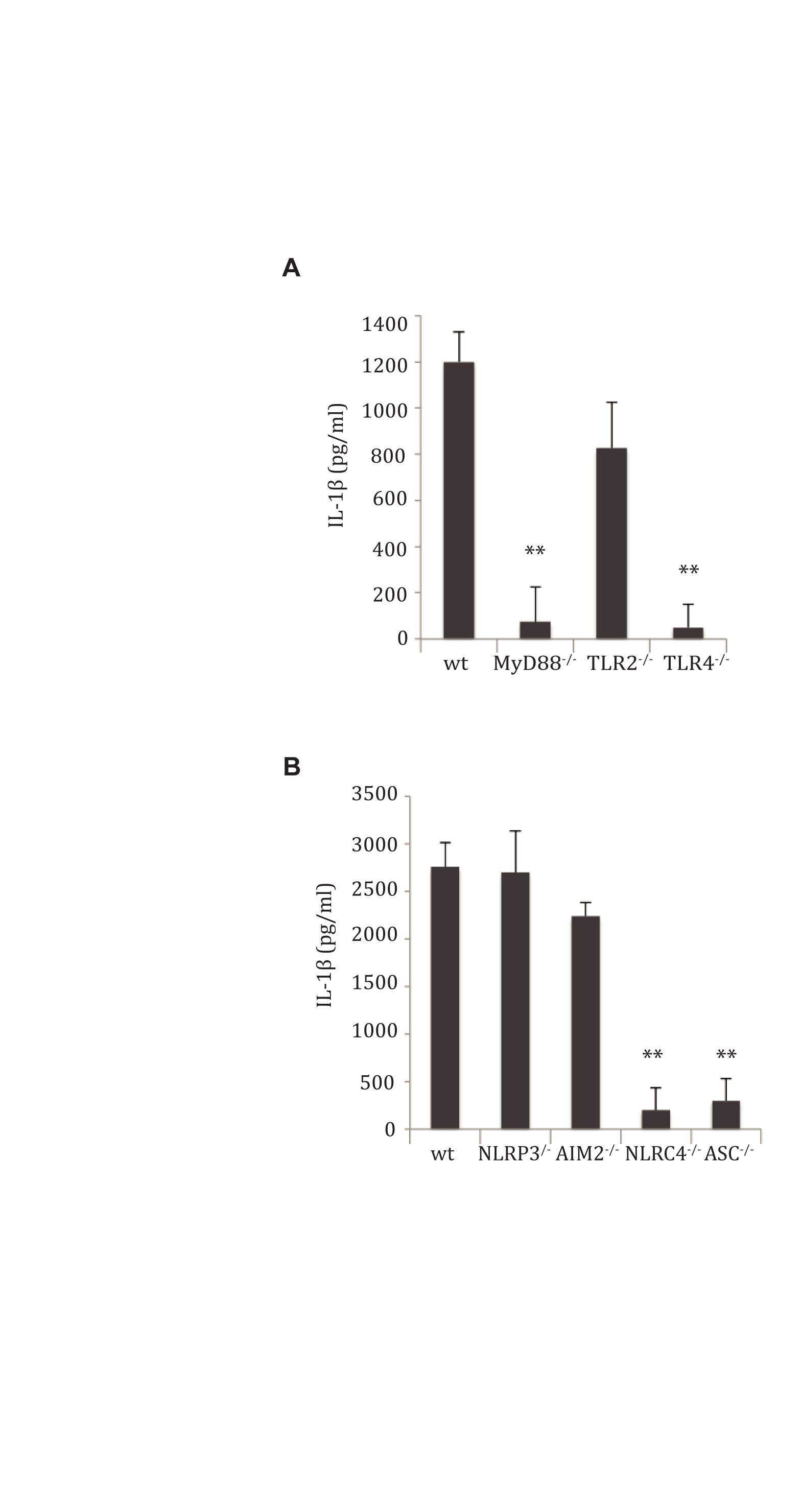

Supplement: S3 Fig — IL-1β ELISA performed on supernatants obtained from BMDMs 24 hpi with Y. pestis strains CO92 at MOI 10. A. BMDMs were derived from age-matched female wild-type, MyD88-/-, TLR2-/- and TLR4-/- mice, and B. NLRP3-/-, NLRC4-/-, AIM2-/-, and ASC-/- mice. (TIF) [file ppat.1004688.s003.tif]

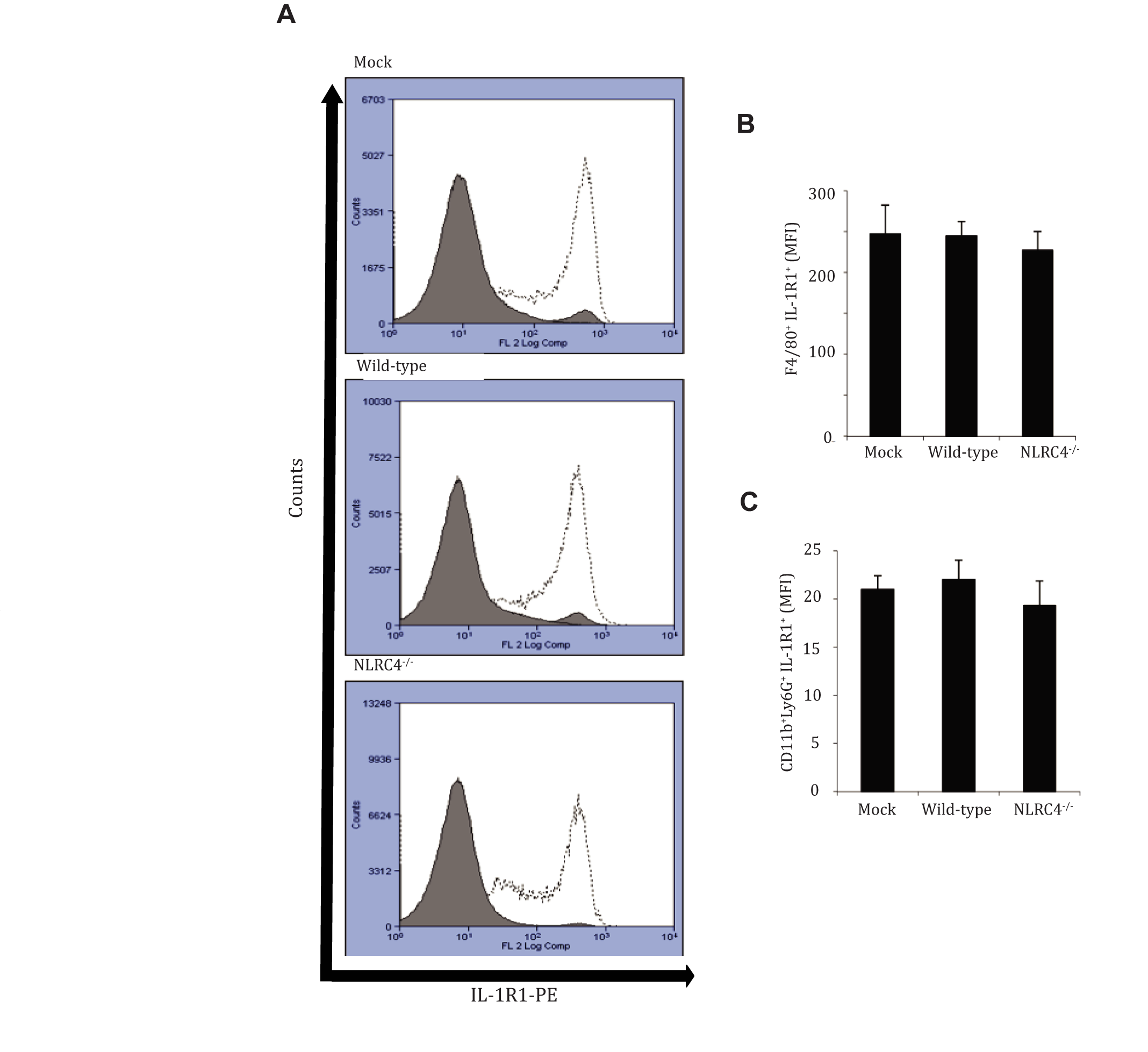

Supplement: S4 Fig — Wild-type and NLCR4-/- mice were intranasally inoculated with Y. pestis and sacrificed at 12 hpi. Total cells were isolated from lungs and flow cytometry was performed to detect IL-1R1 expression on cell types as previously described [4,17]. A. Histograms depict IL-1R1 expression in total cells isolated from mock-infected and Y. pestis-infected wild-type and NLRC4-/- mice. B. Mean Florescence Intensity (MFI) of IL-1R1 expression was measured for both F4/80+ macrophages and C. F4/80- Ly6G+ neutrophils. (TIF) [file ppat.1004688.s004.tif]

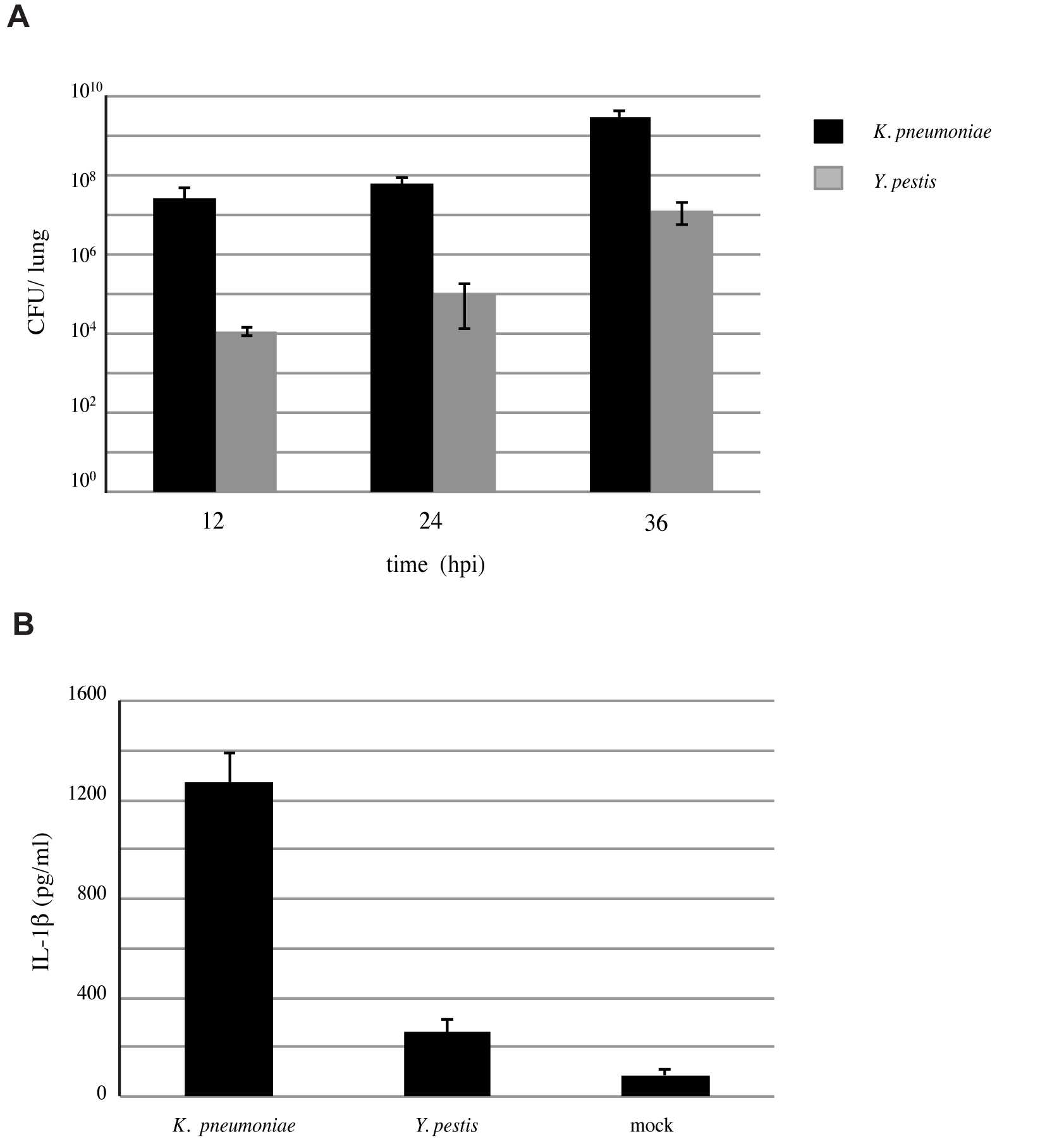

Supplement: S5 Fig — A. Wild-type mice were infected either with Y. pestis (1x10^4^ CFU) or K. pneumoniae (2x10^5^ CFU), and lungs were assessed for bacterial burden at 12, 24 and 36 hpi. B. IL-1β was detected from lung homogenates of mice at 24 hpi. (TIF) [file ppat.1004688.s005.tif]

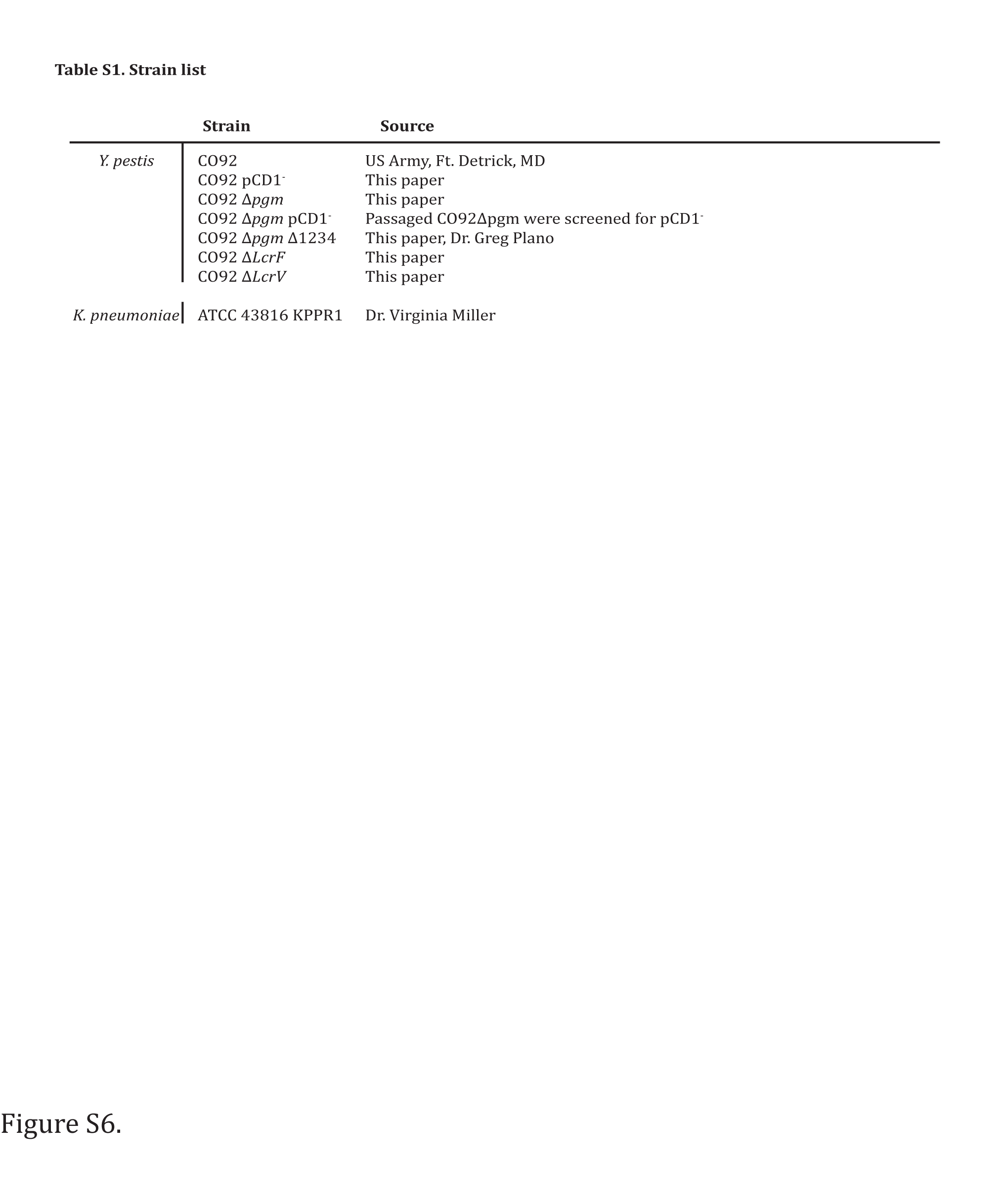

Supplement: S6 Fig — (TIF) [file ppat.1004688.s006.tif]

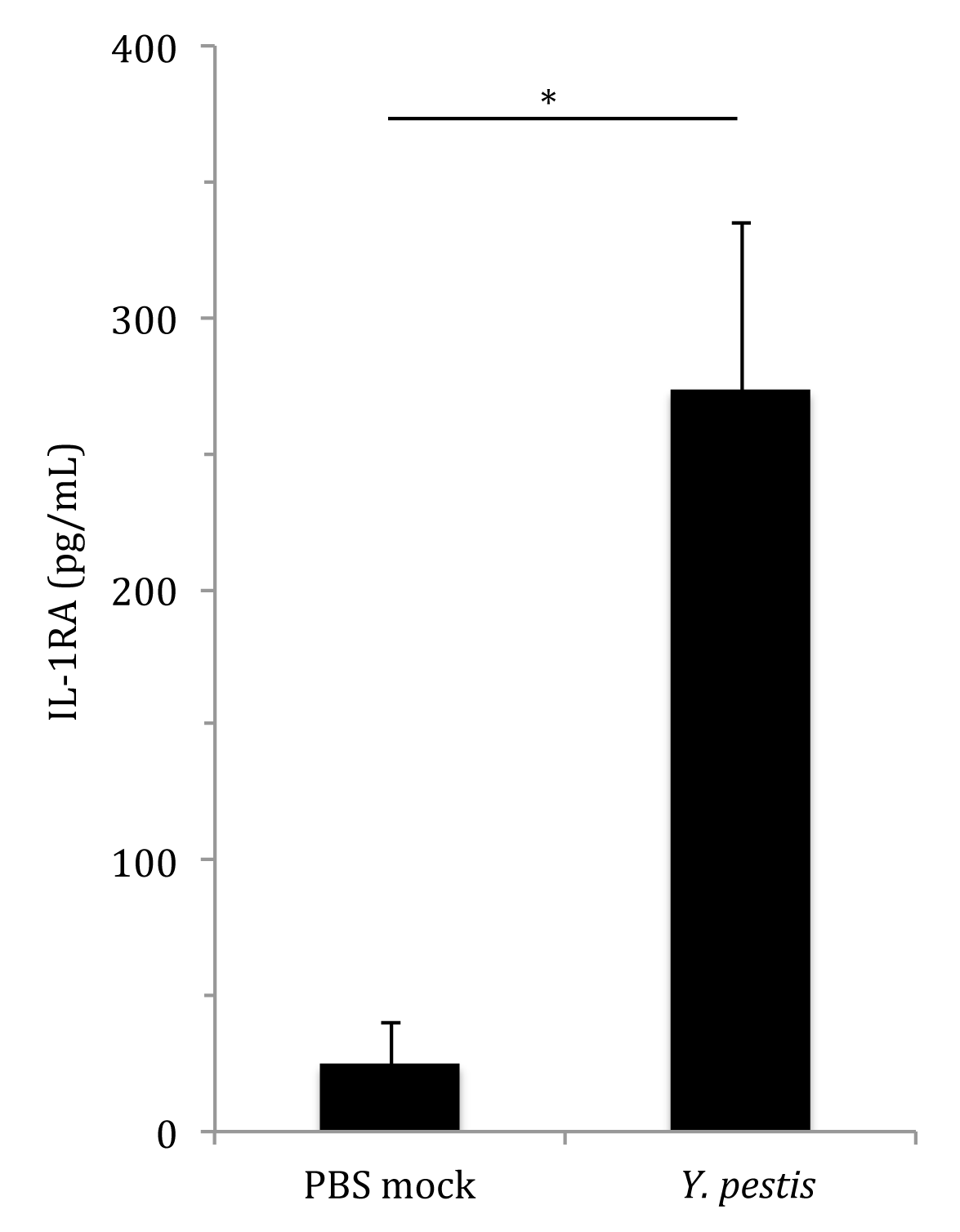

Supplement: S7 Fig — IL-1RA ELISA performed on bronchoalveolar lavage fluid obtained from mice 24 h after inoculation with 104 CFU Y. pestis or PBS (mock-infected). Data represent mean ± SEM from two independent pooled experiments (n = 6-8 mice per group). * p<0.05 by standard t-test. (TIF) [file ppat.1004688.s007.tif]
