# Supplementary material for: Correction: Yersinia pestis Activates Both IL-1β and IL-1 Receptor Antagonist to Modulate Lung Inflammation during Pneumonic Plague
Source: PLoS Pathog. 2015 May 13;11(5):e1004921. doi: 10.1371/journal.ppat.1004921 (PMC4430546; doi:10.1371/journal.ppat.1004921)
Supplement: S2 File — (PDF) [file ppat.1004921.s002.pdf]

PLOS | PATHOGENS

RESEARCH ARTICLE

Yersinia pestis Activates Both IL-1β and IL-1 Receptor Antagonist to Modulate Lung Inflammation during Pneumonic Plague

Vijay Sivaraman[1,2], Roger D. Pechous[1], Nikolas M. Stasulli[1], Kara R. Eichelberger[1], Edward A. Miao[1], William E. Goldman[1]*

1 Department of Microbiology and Immunology, University of North Carolina at Chapel Hill, Chapel Hill, North Carolina, United States of America, **2** Department of Biology, North Carolina Central University, Durham, North Carolina, United States of America

* goldman@med.unc.edu

Abstract

Pneumonic plague is the most rapid and lethal form of *Yersinia pestis* infection. Increasing evidence suggests that *Y. pestis* employs multiple levels of innate immune evasion and/or suppression to produce an early "pre-inflammatory" phase of pulmonary infection, after which the disease is highly inflammatory in the lung and 100% fatal. In this study, we show that IL-1β/IL-18 cytokine activation occurs early after bacteria enter the lung, and this activation eventually contributes to pulmonary inflammation and pathology during the later stages of infection. However, the inflammatory effects of IL-1β/IL-1-receptor ligation are not observed during this first stage of pneumonic plague. We show that *Y. pestis* also activates the induction of IL-1 receptor antagonist (IL-1RA), and this activation likely contributes to the ability of *Y. pestis* to establish the initial pre-inflammatory phase of disease.

Author Summary

Inhalation of respiratory droplets containing *Yersinia pestis* results in a rapidly developing and lethal pneumonia. Interestingly, early interactions between *Y. pestis* and host cells in the lung contribute to significant immune evasion, but also ultimately result in severe innate immune activation. Our results demonstrate that *Y. pestis* activates pro-inflammatory cytokines IL-1β and IL-18 in the lung early during infection. However, there is very little early pulmonary inflammation while *Y. pestis* continues to multiply in the lung compartment. We show that the host protein IL-1RA is activated concurrently with IL-1β, attenuating early immune activation by this cytokine. We propose that this allows the organism to replicate to high titers, eventually triggering a vigorous inflammatory response and facilitating aerosol transmission. Therefore, evaluating early host activation of IL-1RA by *Y. pestis* may provide therapeutic targets against pneumonic plague.

CrossMark
click for updates

OPEN ACCESS

Citation: Sivaraman V, Pechous RD, Stasulli NM, Eichelberger KR, Miao EA, Goldman WE (2015) *Yersinia pestis* Activates Both IL-1β and IL-1 Receptor Antagonist to Modulate Lung Inflammation during Pneumonic Plague. PLoS Pathog 11(3): e1004688. doi:10.1371/journal.ppat.1004688

Editor: David Weiss, Emory University School of Medicine, UNITED STATES

Received: May 21, 2014

Accepted: January 19, 2015

Published: March 17, 2015

Copyright: © 2015 Sivaraman et al. This is an open access article distributed under the terms of the Creative Commons Attribution License, which permits unrestricted use, distribution, and reproduction in any medium, provided the original author and source are credited.

Data Availability Statement: All relevant data are within the paper and its Supporting Information files.

Funding: The described work was funded by National Institutes of Health grants AI057157 and AI099698. The funders had no role in study design, data collection and analysis, decision to publish, or preparation of the manuscript.

Competing Interests: The authors have declared that no competing interests exist.

Introduction

The innate immune system plays an integral role in controlling microbial infection, providing the first layer of defense against invading pathogens and incorporating multiple levels of threat detection. Sentinel cells such as macrophages and neutrophils use pattern recognition receptors (PRRs) that detect pathogen-associated molecular patterns (PAMPs), which are common microbial components such as lipopolysaccharide (LPS) and peptidoglycan. Two major classes of PRRs are Toll-like receptors (TLRs) and NOD-like receptors (NLRs). PRR-mediated recognition of microbial ligands leads to the secretion of pro-inflammatory signals that curtail the growth and spread of pathogenic microbes at the initial sites of infection. Recognition of PAMPs by cell surface or endosomal TLRs results in the activation of pro-inflammatory cytokines including Type 1 interferons, IL-6, TNFα, IL-1β and IL-18. PAMPs that penetrate the host cell cytoplasm are detected by NLRs such as NLRP1, NLRP3, NLRC4, AIM2, NLRC5, NAIP proteins, and NLRP12 [1–7]. Intracellular detection of PAMPs leads to the formation of a complex of proteins known as the "inflammasome." Inflammasome assembly leads to activation of Caspase 1, and ultimately results in the processing and secretion of TLR-primed cytokines IL-1β and IL-18. Secreted IL-1β and IL-18 bind to receptors expressed on the surface of most cells in the body, which leads to cellular NFκB activation and additional pro-inflammatory cytokine production. The ability of a pathogen to successfully establish infection often relies upon its evasion and/or suppression of these multi-layered immune detection systems.

Yersinia pestis is the causative agent of bubonic and pneumonic plague. Pneumonic plague is a rapidly progressing, severe pulmonary infection that can be transmitted by aerosol, leading to the classification of *Y. pestis* as a Tier 1 Select Agent. Work by our group and others have characterized the progression of pneumonic plague using both inbred and outbred mouse models of infection [8,9]. The syndrome manifested in mice closely mirrors human infection, and thus represents an appropriate model for discriminating both microbial and host mediators of disease. Pneumonic plague presents as two distinct phases of disease: an extended "pre-inflammatory" phase during which bacterial replication occurs in the absence of discernible disease symptoms or inflammatory responses, and a subsequent "pro-inflammatory" phase characterized by the onset of symptoms, dramatic increases in lung cytokines, neutrophils, and inflammatory lung pathology. The delayed appearance of symptoms combined with the short time course of disease is a major clinical challenge, and the narrow window for effective antibiotic treatment is largely responsible for mortality rates approaching 100% [10].

Inflammasome activation is important during bacterial infection as it induces pyroptotic cell death, and can determine the course of host inflammatory responses to invading pathogens. It has been shown that *Yersinia* species are able to trigger inflammasome activation, and that *Yersinia* effector proteins can inhibit this activation as well as its downstream effects [1,5,11–13]. We sought to evaluate inflammasome activation in our murine infection model of pneumonic plague to determine if this process is inhibited to facilitate the "immunologically silent" pre-inflammatory phase of disease. In the work presented here, we show that inflammasome activation occurs early during pneumonic plague and ultimately contributes to progression into the pro-inflammatory phase of disease. Further, we suggest that *Y. pestis* induction of host IL-1 receptor antagonist (IL-1RA) is a potential mechanism to maintain the pre-inflammatory state despite inflammasome activation, thus allowing for undeterred bacterial replication in the lung.

Results

Activation of IL-1β/ IL-18 cytokines occurs early during pneumonic plague

To examine IL-1β/IL-18 activation during *Y. pestis* pulmonary infection, we first monitored IL-1β in the lungs of infected mice at various times during the pre-inflammatory phase of disease. Both unprocessed and secreted forms of IL-1β protein were observed as early as 6 hpi and persisted through 24 hpi, as confirmed by Western blot and ELISA analysis of whole lung homogenates (Fig. 1A,B). Similar results were observed for IL-18 induction, albeit with a delayed time of activation (S1 Fig). This is surprising, as we (and others) have demonstrated a notable lack of pro-inflammatory cytokine induction early during pulmonary *Y. pestis* infection [8,9,14]. These data indicate that IL-1β/IL-18 activation in the lung occurs at least 30 hours before any other signs of inflammation in pneumonic plague.

The bacterial type III secretion system (T3SS) has been shown to play a role in IL-1β/IL-18 cytokine activation for a number of pathogens, including *Yersinia* species [1,4,15,16]. In some cases aberrant secretion of T3SS components was responsible, and for some organisms insertion of the secretion apparatus itself initiated activation of IL-1β/IL-18 cytokines [4]. We sought to investigate the requirement of this system for activation of IL-1β in response to infection with *Y. pestis*. To this end, we tested whether *Y. pestis* carrying the genes encoding the injection apparatus but lacking the Yop effector proteins (designated as CO92 Δ*pgm* Δ1234) was able to activate IL-1β during pneumonic plague. IL-1β protein was observed in mouse total lung homogenates at 24 hpi with the parent CO92 Δ*pgm* and CO92 Δ*pgm* Δ1234 strains, but much less IL-1β was seen in animals infected with the CO92 Δ*pgm* pCD1$^-$ strain lacking the T3SS genes (Fig. 1C). This was not due to differential microbial replication, as mean bacterial burdens were relatively equivalent between the three groups (Fig. 1D). We have previously reported that *Y. pestis* preferentially targets alveolar macrophages for injection of type III effectors within the lung at early times during infection [17]. We therefore recapitulated this phenomenon *in vitro* with infection of BMDMs. Our results confirmed that *Y. pestis* induces IL-1β secretion via a mechanism that requires an intact T3SS (S2 Fig). These data demonstrate that the type III secretion apparatus is required for optimal *Y. pestis*-mediated activation of IL-1β. Further, this activation occurred in the absence of all of the Yop effector proteins, indicating that the needle apparatus itself is sufficient for activating IL-1β during pneumonic plague.

Inflammasome activation contributes to the pathogenesis of pneumonic plague in a TLR4/NLRC4-dependent manner

IL-1β and IL-18 secretion is a key marker of inflammasome assembly and activation. Inflammasome activation is an important mediator of host inflammatory responses and has been shown to be important to controlling infection with a number of pathogens, primarily through the secretion of IL-1β and IL-18 [5,18–20]. To determine if IL-1β and IL-18 activation plays a role in controlling *Y. pestis* infection, we infected IL-1β/IL-18$^{-/-}$ and wild-type C57BL/6 mice with a lethal dose (10^4 CFU) of fully virulent *Y. pestis* strain CO92 and monitored survival, bacterial burden and lung histopathology. In addition, we infected Caspase 1$^{-/-}$ mice to investigate the role of inflammasome activation in controlling *Y. pestis* infection. Mice lacking either Caspase 1 or the inflammasome cytokines IL-1β and IL-18 demonstrated extended survival compared to wild-type C57BL/6 mice (Fig. 2A), suggesting that IL-1β and IL-18, and likely inflammasome activation, contribute to the rapid mortality seen during pneumonic plague. This is surprising, as we expected that the absence of IL-1β and IL-18 cytokine activation might accelerate time-to-death due to the impairment of this frontline innate immune defense

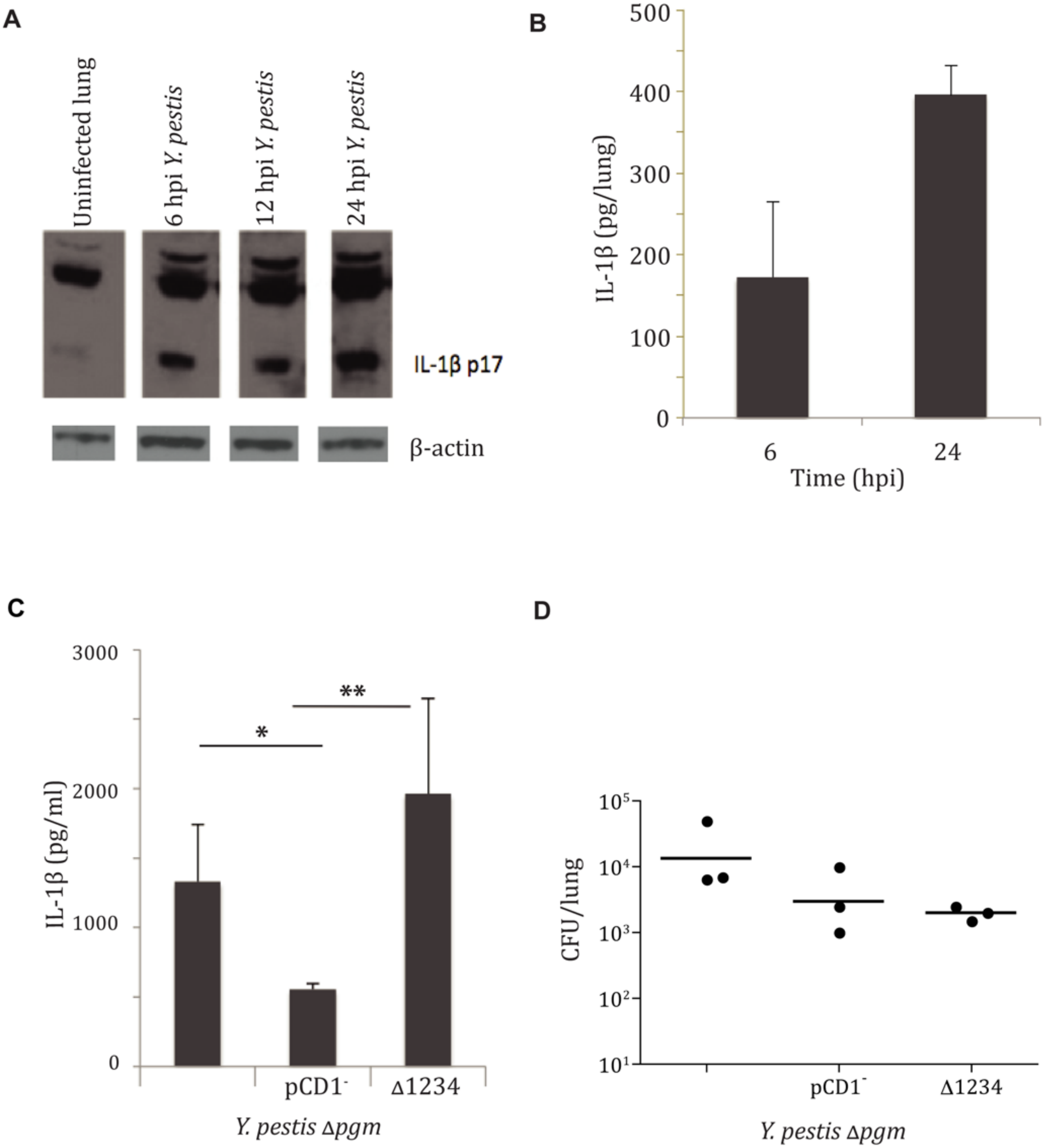

Fig 1. *Yersinia pestis* type III injectisome activates IL-1β and IL-18 cytokines early during lung infection. A. IL-1β Western blot analysis performed on total lung homogenate harvested at 6, 12, and 24 hpi with LD_{100} infection of *Y. pestis* strain CO92, compared to uninfected mice. **B.** IL-1β ELISA performed on total lung homogenates harvested at 6 and 24 hpi. **C.** Bacterial burdens from lungs of mice 24 hpi with 10^4 CFU/ mouse with the strains mentioned (denoted by T3SS) or parent stain (unlabeled). **D.** IL-1β ELISA performed on total lung homogenates harvested at 24 hpi with with the strains mentioned. All infections were performed in triplicate, with representative analysis shown. * $p< 0.05$, ** $p<0.001$.

doi:10.1371/journal.ppat.1004688.g001

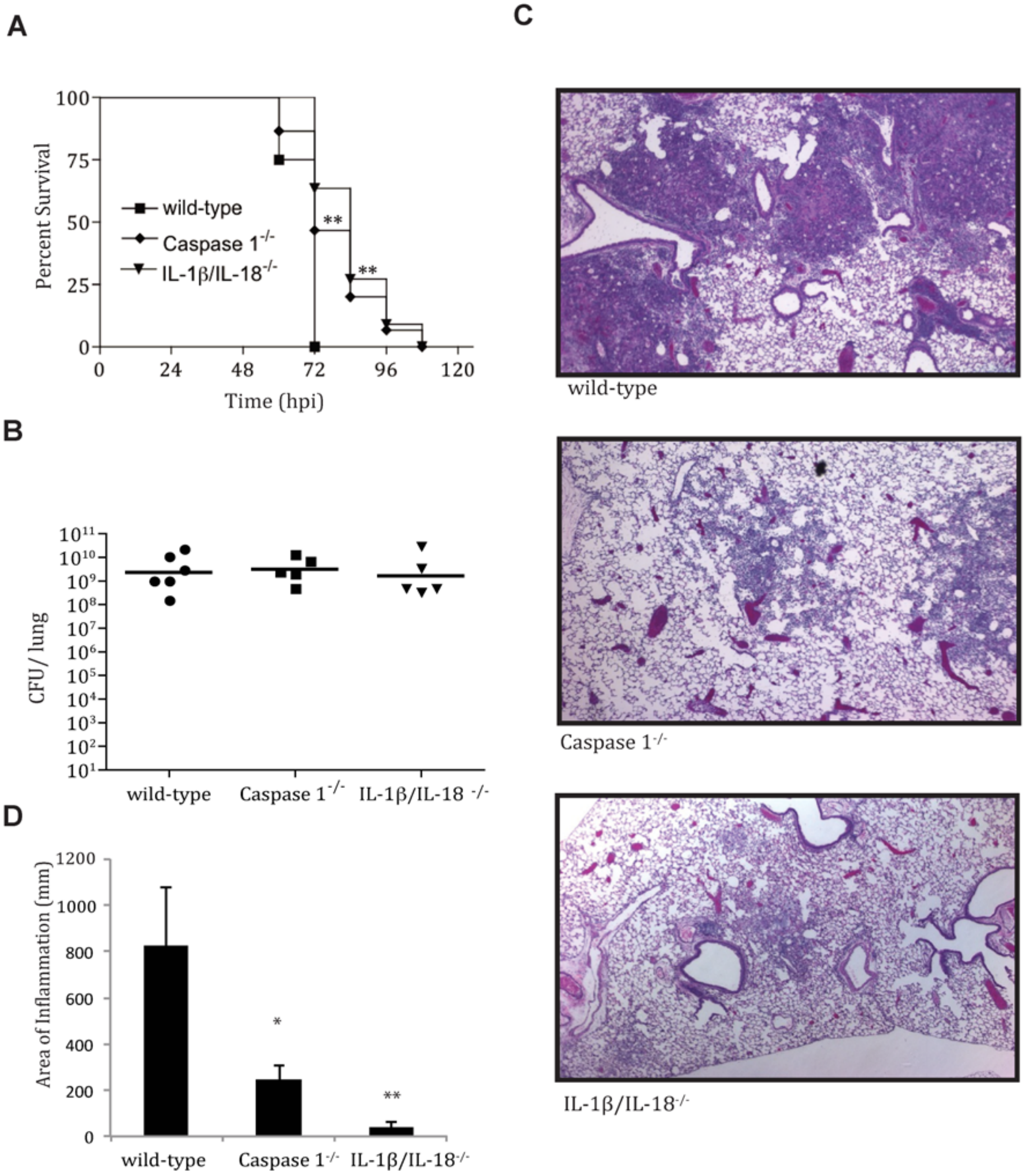

Fig 2. IL-1β/IL-18 cytokine activation contributes to pathology of pneumonic plague. A. A survival analysis was performed comparing wild-type (n = 8), Caspase $1^{-/-}$ (n = 15) and Il-1β/IL-$18^{-/-}$(n = 12) mice (on C57BL/6 background) after LD_{100} infection (10^4 CFU/mouse) of *Y. pestis* strain CO92. **B.** Total lung burden (CFU/lung) at 48 hpi after LD_{100} infection with *Y. pestis* strain CO92. **C.** fixed H&E stained lung sections from mice at 48 hpi. D. Inflammation from histopathology sections was quantified as described in Methods. * $p<0.05$, ** $p<0.001$.

doi:10.1371/journal.ppat.1004688.g002

mechanism. In addition, mice lacking Caspase 1 or mice lacking IL-1β and IL-18 had markedly decreased symptoms of respiratory distress in response to *Y. pestis* infection. This was not due to differences in bacterial numbers, since bacterial burdens within the lungs of wild-type mice, mice deficient in Caspase 1, and mice deficient in IL-1β/IL-18 were equivalent (Fig. 2B). H&E staining of lungs from Caspase 1-deficient mice or IL-1β/IL-18-deficient mice demonstrated decreased inflammatory lesion formation compared to wild-type mice (Fig. 2C,D). These data

suggest that Caspase 1-mediated IL-1β and IL-18 activation contributes to the severe lung pathology and enhanced mortality seen during pneumonic plague, but does not contribute to host control and/or clearance of *Y. pestis* during pneumonic plague.

Inflammasome complex assembly is described as a two-signal system: The primary signal requires TLR activation that leads to transcription of pro-IL-1β and IL-18, whereas the secondary signal requires detection of microbial ligands by NLRs to assemble the NLR/ASC/Caspase 1 complex that proteolytically cleaves and activates the pro-forms of IL-1β and IL-18 for secretion. The decreased inflammation seen in Caspase $1^{-/-}$ mice indicates that inflammasome activation contributes to the progression of pneumonic plague. To identify the host signals responsible for *Y. pestis* activation of the inflammasome, we inoculated macrophages derived from $TLR2^{-/-}$, $TLR4^{-/-}$ and $MyD88^{-/-}$ mice with the fully virulent strain *Y. pestis* CO92. TLR2 recognizes a variety of PAMPs including peptidoglycan and lipoproteins, the TLR4 receptor recognizes bacterial lipopolysaccharide (LPS), and MyD88 serves as a common intermediate signaling protein downstream of most TLRs. We observed that IL-1β secretion is not dependent on TLR2, but requires the presence of TLR4 and MyD88 (S3A Fig). These data were confirmed in $TLR4^{-/-}$ mice inoculated with *Y. pestis*: IL-1β production (Fig. 3A) as well as downstream pulmonary inflammation (Fig. 3B) both indicated that TLR4 contributes to IL-1β secretion in vivo. Also, though recent studies have shown that some microbial/host cell interactions lead to a Caspase 1-independent IL-1β activation, we observed that Caspase 1 is necessary for early IL-1β activation (Fig. 3C)[21,22]. These data indicate that *Y. pestis* secretes IL-1β via TLR4 and Caspase 1-dependent mechanisms. This is interesting, as *Y. pestis* LPS is tetra-acylated at 37°C and is therefore significantly less immunostimulatory than that of organisms with the more typical hexa-acylated LPS known to bind TLR4 [23].

Of the growing NLR protein family, NLRP1, NLRP3, NLRC4 and AIM2 are known to associate with Caspase 1 to form an inflammasome. Previous literature indicates that $NLRP3^{-/-}$ is primarily responsible for *Y. pestis* inflammasome activation *in vitro* [7]. To identify the host mediators of inflammasome activation during pneumonic plague, we examined IL-1β production and *Y. pestis* virulence within the lungs of $NLRP3^{-/-}$, $NLRC4^{-/-}$ and wild-type mice. At 24 hpi, IL-1β activation was observed in the lungs was observed in the lungs of both wild-type and $NLRP3^{-/-}$ mice, but not in $NLRC4^{-/-}$ mice (Fig. 3C). Inflammatory lesion formation was diminished in $NLRC4^{-/-}$ mouse lungs, but was similar between $NLRP3^{-/-}$ and wild-type mice (Fig. 3D,E). However, *Y. pestis* replication in the lung was unhindered in the absence of either NLRP3 or NLRC4 (Fig. 3F), thus decoupling bacterial burden from pulmonary inflammation. In summary, these data suggest that *Y. pestis* primes specific cytokines and activates the inflammasome within the lung through both TLR4 and NLRC4, and this early inflammasome activation plays a significant role in the progression to the pro-inflammatory phase of disease.

IL-1 receptor antagonist protein and IL-18 binding protein are induced by *Y. pestis* early during infection

Early inflammasome activation by *Y. pestis* is paradoxical: our data show that early inflammasome activation contributes to inflammation in the lung and enhanced mortality, but lung inflammatory responses are delayed at least 30 hours after pro-inflammatory IL-1β secretion is detected. We sought to determine the molecular explanation for this extended pre-inflammatory phase of disease. Both macrophages and neutrophils express the IL-1 receptor (IL-1R1) on their surface and are the primary targets of the *Y. pestis* T3SS early during infection [17]. Secreted IL-1β activates self and neighboring cells through autocrine and paracrine ligation of IL-1R1, leading to early innate immune activation and pulmonary inflammation [3]. We sought to determine whether *Y. pestis* infection in the lung preferentially down-regulates IL-1

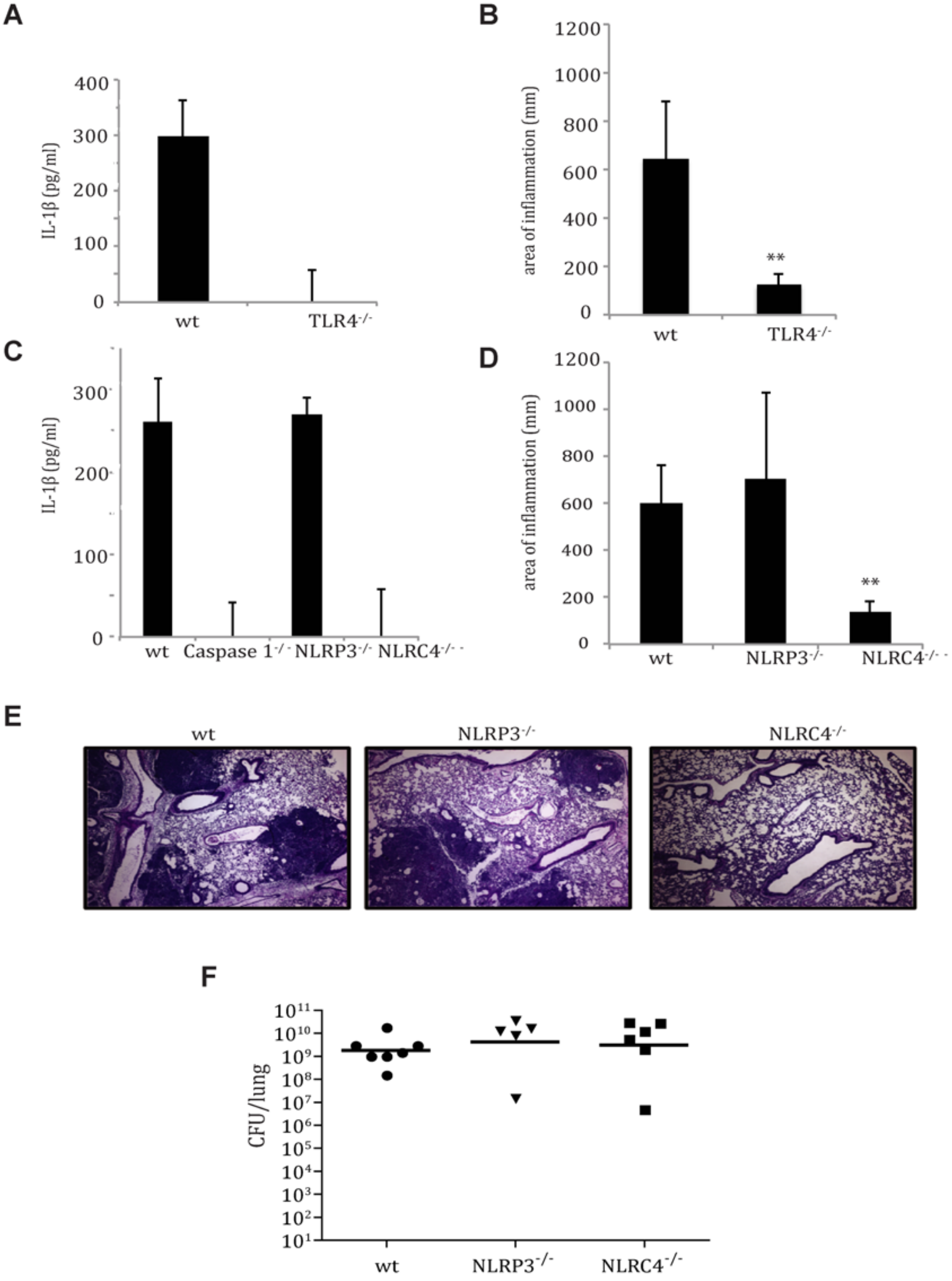

Fig 3. TLR4- and NLRC4-dependent IL-1β and IL-18 activation by *Y. pestis* contributes to respiratory pathology. A. IL-1β ELISA performed on lung homogenates obtained from wild-type and TLR4$^{-/-}$mice, 24 hpi with LD_{100} dose of *Y. pestis*. **B**. Inflammation from histopathology sections from wild-type and TLR4$^{-/-}$ mice 48 hpi with *Y. pestis* was quantified as described in Methods. C. IL-1β ELISA performed on lung homogenates of wild-type, Caspase 1$^{-/-}$, NLRP3$^{-/-}$ and NLRC4$^{-/-}$ mice, 24 hpi with LD_{100} dose of *Y. pestis*. D. Inflammation from histopathology sections from wild-type, NLRP3$^{-/-}$ and NLRC4$^{-/-}$ mice 48 hpi with *Y. pestis*. (N = 5–8 mice/group). **E.** Histology of fixed H&E stained lung sections from mice at 48 hpi. **F.** Bacterial burdens from lungs of age-matched female wild-type, NLRP3$^{-/-}$, and NLRC4$^{-/-}$ mice infected intranasally with LD_{100} dose of *Y. pestis* strain CO92 for 48 hrs. * $p<0.05$, ** $p<0.001$.

doi:10.1371/journal.ppat.1004688.g003

receptors. We did not observe differences in IL-1R1 surface expression between mock and infected mice or with infected NLRC4$^{-/-}$ mice (S4 Fig), indicating that downregulation of IL-1 receptors is likely not a mechanism for maintaining the pre-inflammatory phase of disease.

The IL-1 Receptor Antagonist (RA) and IL-18 Binding Protein (BP) are decoy proteins generated by innate immune cells to dampen inflammatory responses, typically in response to tissue damage. IL-1RA has significant homology to IL-1β but does not have a signaling domain, and is thus non-inflammatory when bound to the cognate IL-1 receptor [24]. IL-18BP binds to the cytokine IL-18 and inhibits its ability to bind to the cognate receptor present on epithelial cells, T cells and NK cells, thus inhibiting downstream pro-inflammatory cytokine activation [25]. It has been shown that *Yersinia* species can activate the expression of IL-1RA in vivo in the lymph nodes and Peyer's patches of infected mice [26–28]. We sought to determine if *Yersinia* induced either IL-1RA or IL-18BP production in the lung, and whether this might contribute to the maintenance of the pre-inflammatory disease phase despite clear inflammasome activation. As a comparison, we also evaluated lungs from animals inoculated with *Klebsiella pneumoniae*, which is known to cause severe pneumonia with early pulmonary inflammation [27–30]. As early as 12 hpi with *Y. pestis*, IL-1RA gene transcription was 12-fold higher than mock-infected animals and remained at that level for both the 24 hpi and 36 hpi time points (Fig. 4A). Early up-regulation of IL-1RA was also evident at the protein level in bronchoalveolar lavage fluid, which showed an increase of IL-1RA protein by approximately 11-fold at 24 hpi with *Y. pestis* (S7 Fig). IL-18BP gene transcription was increased 10-fold at 12 hpi, but expression substantially decreased at later time points (Fig. 4B). Pulmonary infection with *K. pneumoniae* did not upregulate transcription for either of these genes until 36 hpi despite greater bacterial burdens (S5A Fig), indicating that this early induction is not common to all inflammatory respiratory bacteria. This is interesting, as IL-1β was significantly activated in the lungs of *K. pneumoniae*-inoculated animals compared to animals inoculated with *Y. pestis* at 24 hpi (S5B Fig). In summary, *Y. pestis* infection leads to robust and sustained up-regulation of the anti-inflammatory protein IL-1RA, concurrent with early inflammasome activation.

IL-1RA suppresses early immune activation to enhance bacterial replication

Given that the expression of the IL-1RA gene was upregulated early and sustained in the lung after inoculation with *Y. pestis*, we sought to examine whether this protein aided in early host suppression of innate immune responses by *Y. pestis* during pulmonary infection. We hypothesized that depletion of this protein would lead to an early inflammatory response capable of limiting bacterial replication. Mice were administered IL-1RA neutralizing antibody (IL-1RA nAb) intranasally, and then inoculated with a lethal dose of *Y. pestis* strain CO92. In mice treated with IL-1RA nAb, small foci of inflammatory cells were visible within lungs at 24 hpi, while animals treated with isotype control antibody had no visible inflammation present (Fig. 5A,B). At the same time, lung bacterial burden decreased by more than 10-fold in animals treated with IL-1RA neutralizing antibody (nAb) compared to isotype control antibody- treated animals at 24 hpi (Fig. 5C). Importantly, we observed an early increase in pro-inflammatory cytokines TNFα, IFNγ, IL-6 and IL-12 in animals treated with IL-1RA nAb at 24 hpi (Fig. 5D). These data suggest that IL-1RA neutralization may provide IL-1 receptor access for activation, resulting in early inflammation in the lung. In summary, these data present a scenario where *Y. pestis* induces IL-1RA in order to partially suppress early host inflammatory responses and colonize the lungs more efficiently during the pre-inflammatory phase of pneumonic plague.

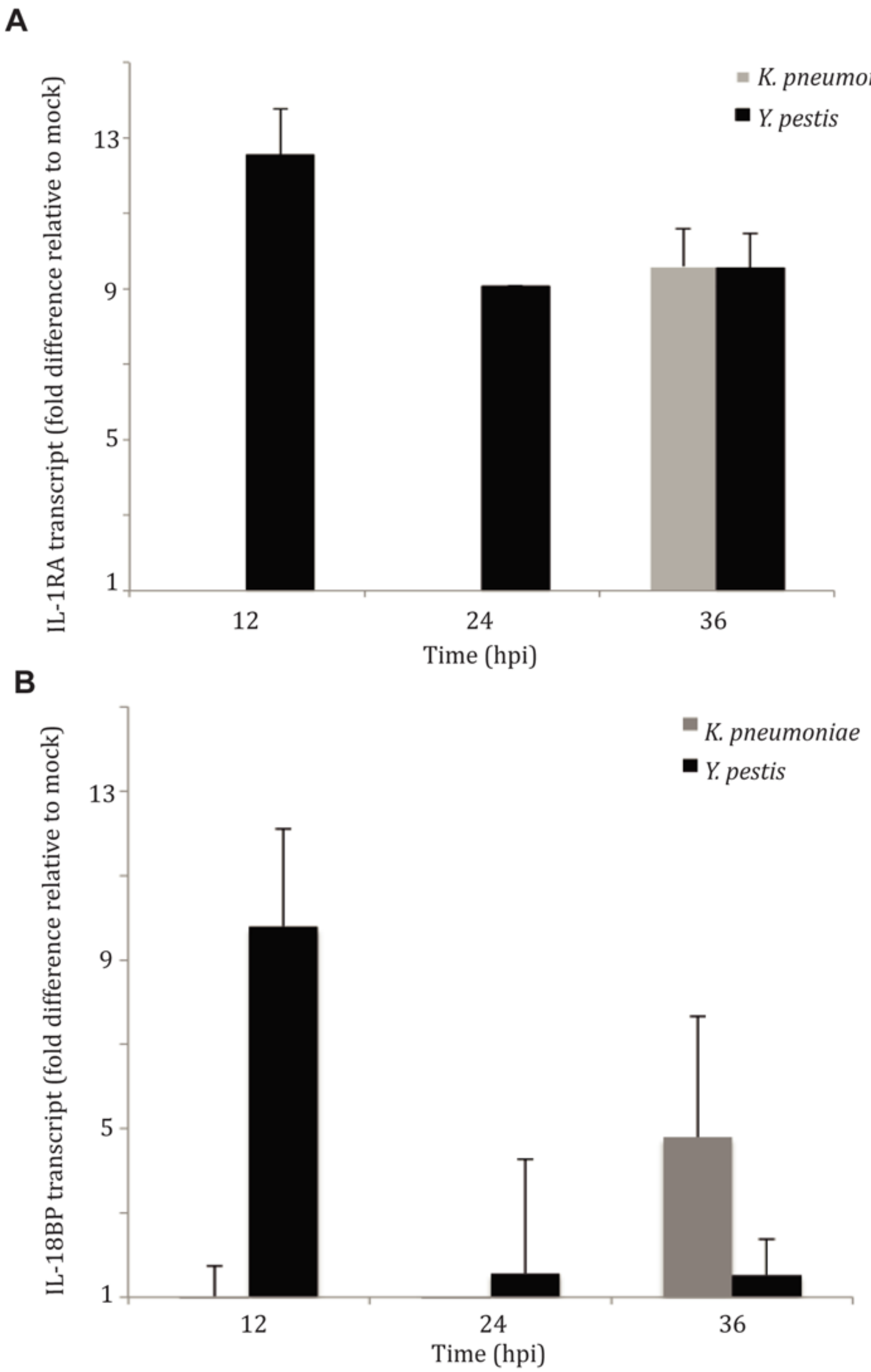

Fig 4. IL-1RA is induced in the lung early during pulmonary infection with *Y. pestis*, but not *K. pneumoniae*. Real time qRT-PCR analysis for (**A**) IL-1RA and (**B**) IL-18BP activation from lungs of mice inoculated with either an LD_{100} dose of *Y. pestis* ($1x10^4$ CFU) or *K. pneumoniae* ($2x10^5$ CFU), at 12, 24 and 36 hpi (N = 3, per experiment). Fold difference calculated as $\Delta\Delta$ C_t.

doi:10.1371/journal.ppat.1004688.g004

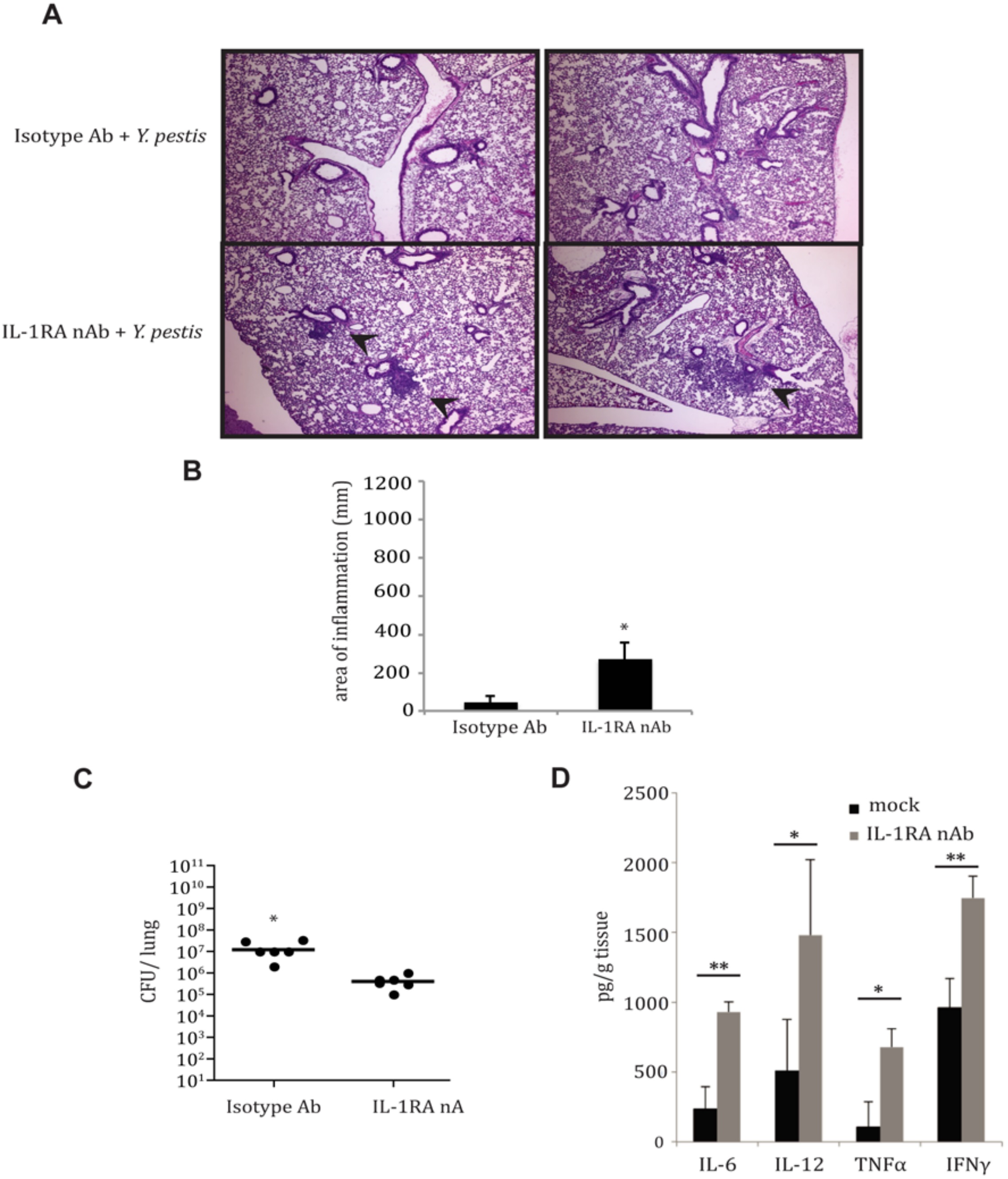

Fig 5. IL-1RA contributes to control of *Y. pestis* replication and pathology in the lung. A. Histology of fixed H&E stained lung sections from mice at 24 hpi with *Y. pestis*, +/- IL-1RA nAb. Small foci of inflammation indicated by arrows. **B.** Inflammation from histopathology sections was quantified as described in Methods. **C**. Bacterial burdens were assessed from mice 24 hpi with *Y. pestis*, +/- IL-1RA nAb. **D**. Cytokine analysis from lysates of homogenized lungs of mice infected with *Y. pestis* +/- IL-1RA nAb for 24 hrs was perfomed by ELISA (BD Pharmingen). * $p<0.05$, ** $p<0.001$.

doi:10.1371/journal.ppat.1004688.g005

Discussion

Pneumonic plague develops in two distinct stages, beginning with an extended "pre-inflammatory phase." During this period, *Y. pestis* transforms localized areas of the lung into a permissive environment that allows for significant bacterial growth, and has been characterized as a

dominant immunosuppressive state [28]. In the current study we demonstrate that *Y. pestis* activates the inflammasome early during the pre-inflammatory phase, triggering IL-1β secretion in the lung. This is in contrast to a myriad of other pro-inflammatory cytokines that are not induced until the later stages of infection. Though inflammasome activation has a protective effect against a number of pathogens [20,30,31], we show that the inflammasome contributes to pulmonary pathology and lethality during pneumonic plague. Our data also suggest that *Y. pestis* infection concomitantly stimulates production of the anti-inflammatory protein IL-1RA, which may explain how the pre-inflammatory phase can be maintained despite inflammasome activation. This may be an important mechanism to suppress host immune responses, allowing for enhanced *Y. pestis* colonization of the lung.

We observed that *Y. pestis* is able to activate the inflammasome through host detection of the T3SS injectisome, even in the absence of secreted Yops (Fig. 1). Host detection of microbial T3SS systems by NLRs in the absence of secreted effectors can occur by recognition of aberrantly secreted "needle" protein or through pore formation in the host cell outer membrane [4]. We show that *Y. pestis* activation of the inflammasome occurs primarily though an NLRC4-dependent pathway that also involves TLR4. Previous work has shown the tetra-acylation of *Y. pestis* LPS impedes TLR4 activation for a number of inflammatory cytokines, yet the inflammasome cytokines are still primed within lung macrophages [23]. This may be a result of direct interactions between the T3SS needle subunit protein and TLR4, as highlighted recently by Jessen et al [32]. The *Y. pestis* T3SS injectisome activation of NLRC4 corroborates studies with other respiratory pathogens [18,20], and this specificity of activation demonstrates yet another layer of tight control of immune activation.

Recent studies with in vitro and in vivo models of infection have demonstrated activation of IL-1RA by *Y. pestis* [26,28]. Previous work has demonstrated the ability of IL-1RA to attenuate the severity of asthma and ventilator-induced lung injury [33–35], but a role in pulmonary microbial infection has not been previously demonstrated. In the work presented here, we describe a potential effect of IL-1RA activation by *Y. pestis* in the lung. In the case of pneumonic plague, *Y. pestis* induces IL-1RA during the pre-inflammatory phase of disease, thus limiting inflammatory cytokine induction early during infection. Inflammasome activation, though triggered early, does not have pathological consequences until much later, when the bacterial number is extremely high and the disease progresses to the pro-inflammatory stage. At that point, *Y. pestis* activation of the inflammasome is clearly requisite for the observed lung damage and rapid mortality, as demonstrated by the reduced pulmonary inflammation and enhanced survival observed in Caspase $1^{-/-}$ and IL-1β/IL-$18^{-/-}$ mice. Our data suggests that *Y. pestis* activates both the inflammasome as well as IL-1RA to dynamically regulate immune activation, allowing for enhanced bacterial burden early within the lung. This may be favorable for disease, as hyper-immune activation later during infection may result in greater pulmonary damage, coughing, and enhanced transmission.

There are a variety of studies, with sometimes disparate conclusions, regarding inflammasome activation during *Yersinia* infection. The ability of *Y. pestis* to activate the inflammasome is well-documented, and it has been shown that this activation and its downstream effects can be inhibited by various *Yersinia* effectors including YopM and YopK [1,11]. This is the first example of *Y. pestis* inflammasome activation in the lung during pneumonic plague. Surprisingly, this activation occurred early during the "immunologically silent" pre-inflammatory phase of disease, thus requiring an explanation as to why the downstream effects of this activation are delayed. We hypothesize that IL-1RA plays a role in facilitating the pre-inflammatory phase by modulating the ability of the host to respond to *Y. pestis* activation of innate immune mediators.

If the pathology observed during *Y. pestis* respiratory infection requires inflammasome activation, several therapeutic options may be useful for treating human infection. Our work demonstrates that host protein NLRC4 is the primary mediator of *Y. pestis* injectisome detection by the host. Intriguingly, several other Gram-negative pathogens including *P. aeruginosa* have been shown to activate the inflammasome through the NLRC4 complex to enhance pathology [16]. Therefore, NLRC4 may represent a multipotential target for prophylactic drugs. As mentioned earlier, the timing of the pneumonic plague syndrome—a long delay before symptoms followed by the rapid progression of severe pulmonary inflammation—creates a formidable clinical dilemma: by the time a patient is diagnosed with pneumonic plague, it is often too late to administer antibiotics effectively. Current experiments are underway to examine whether treatment with the IL-1RA analog anakinra can reduce respiratory inflammation and reduce pulmonary pathology in *Y. pestis*-infected animals, extending the window for antibiotic treatment and reducing the high mortality of pneumonic plague.

Materials and Methods

Ethics statement

The use of vertebrate animals was performed in accordance with the Public Health Service (PHS) policy on Humane Care and Use of Laboratory Animals, the Amended Animal Welfare Act of 1985, and the regulations of the United States Department of Agriculture. All animal studies were approved by the University of North Carolina at Chapel Hill Office of Animal Care and Use, protocols #09–057.0 and #12–028.0.

Strains and culture conditions

Both the virulent and the pCD1$^-$ (avirulent) *Y. pestis* CO92 strains were obtained from the US Army, Ft. Detrick, MD. The presence or absence of pCD1, pMT1, pPCP1 and the *pgm* locus was confirmed by PCR for each strain before use. Y. *pestis* was routinely grown on Brain Heart Infusion (BHI) agar at 26°C for 2–3 days. Cultures were grown in 10 ml of BHI media supplemented with 2.5 mM $CaCl_2$ at 37°C overnight with constant shaking at 250 rpm, conditions conferring expression of genes present on the pCD1 virulence plasmid. *Klebsiella pneumoniae* subspecies *pneumoniae* was obtained as a kind gift from Dr. Virginia Miller (University of North Carolina, at Chapel Hill) and was routinely grown on BHI agar at 26°C for 1 day or in BHI media at 37°C overnight with constant shaking at 250 rpm. The CO92 Δ1234 strain was constructed as follows: Plasmid CC581 (pCD1 Δ1234) was obtained as kind gift from Dr. G. Plano (University of Miami) and electroporated into *Y. pestis* CO92 *pgm*$^-$ pCD1$^-$, then selected in the presence of kanamycin and chloramphenicol. A list of all bacterial strains used in this study appears in S6 Fig.

Construction of *Y. pestis* deletion strains

Y. pestis mutant strains were constructed using a modified Lambda red recombination system previously described by Lathem et al (Ref). Briefly, 500 base pairs upstream and downstream of each gene were amplified by PCR. The respective PCR products were gel-purified and combined in splicing by overlap extension (SOE)-PCR reactions with a kan^r cassette flanked by FRT sites, which have been previously amplified from the plasmid pHD13. The PCR products were gel-purified and transformed into a *Y. pestis* strain carrying pWL204, which had been grown at 26°C in the presence of arabinose to induce Lambda red recombinase genes. Putative recombinants were selected on BHI plates containing kanamycin and cured of pWL204 on BHI plates containing 5% sucrose.

Bone marrow-derived macrophage preparation and infection

Murine bone marrow-derived macrophages (BMDMs) were prepared by maturing fresh femur-isolated bone marrow cells for 7 days in the presence of GM-CSF-containing supernatant from L929 cells. Cells were verified for macrophage phenotype using flow cytometry analysis of F4/80 and CD11b expression. For infection experiments, cells were plated at $1x10^6$ cells per well, incubated for 24 hr, and then infected with *Y. pestis* (pre-grown at 37°C in the presence of $CaCl_2$ to induce the virulence plasmid) at a multiplicity of infection (MOI) of 10. After 1 hr of incubation at 37°C, media was removed, cells were gently washed with PBS, and 0.5 ml fresh media was added back for 12–14 hrs at 37°C. Supernatants were harvested and filtered through 0.22 micron filters to removed infectious particles.

Animals and infections

Pathogen-free C57BL/6J mice were obtained from the Jackson Laboratory. $AIM2^{-/-}$ mice were obtained as a kind gift from Dr. Jenny Ting (University of North Carolina at Chapel Hill), and $TLR2^{-/-}$, $TLR4^{-/-}$ and $MyD88^{-/-}$ mice were obtained as a kind gift from Dr. Lola Stamm (The University of North Carolina at Chapel Hill). Animals were housed in high efficiency particulate air-filtered barrier units kept inside a satellite air-flow system within a BSL3 facility for the duration of experiments. Mice were given food and water and maintained at 25°C with alternating 12-hour periods of light and dark. As previously described, bacterial cultures were grown in BHI broth as described above, washed once in sterile phosphate buffered saline (PBS), and maintained at 37°C for intranasal inoculation. Mice were lightly anesthetized and inoculated by the intranasal route with 20 μl of *Y. pestis* at a (LD_{100}) dose of $1x10^4$ CFU, and *K. pneumoniae* at a (LD_{100}) dose of $2x10^5$ CFU in PBS. In the short time-course experiments with Δ*pgm* strains, mice were inoculated with 10^6 CFU as the lack of the *pgm* locus limits bacterial growth in vivo. Actual numbers of colony forming units (CFU) inoculated were determined by plating serial dilutions onto BHI agar. Animals that were moribund (unmoving, diminished or heavily labored breathing) were humanely euthanized with an overdose of phenobarbital sodium (150 mg/kg). Mouse experiments were performed at least 3 times, with N = 5–8 mice.

IL-1RA nAb and IL-18BP nAb reagents and treatment

Mouse IL-1RA/IL-1F3 antibody and Mouse IL-18BP antibody were commercially obtained (R&D Systems) and resuspended in 1X PBS. After administration of anesthesia, 10 ug/mouse of each antibody or isotype control was administered through the intranasal route, 30 minutes post-inoculation with *Y. pestis*.

Histopathology

Groups of three to five mice were inoculated with $1x10^4$ CFU *Y. pestis*. Mice were sacrificed at 48 hpi by overdose of phenobarbital sodium, and the lungs were inflated with 10% neutral buffered formalin through a cannulated intra-tracheal route. The lungs were then removed and suspended in formalin for 3 days in the BSL3 lab pending verification that all samples were negative for bacterial growth. The lungs were then washed with PBS before embedding in paraffin. Five-micrometer sections were stained with hematoxylin and eosin before being examined. All microscopy images were captured using the 4X objective of an Olympus BX60 microscope.

Histopathology scoring of inflammation

The lungs of mice inoculated with 10^4 CFU *Y. pestis* CO92 were harvested at 48 hpi and processed for H&E staining. Lung sections showing inflamed regions were analyzed to calculate

the area occupied by inflammatory foci using ImageJ software. Datarepresent the area (mm^2) of inflammation per field in 3 sections from at least five mice. The mean inflamed area from a total of 20 fields are shown. Asterisks indicate a significant difference in area between sample conditions. * $p<.001$, ** $p<.0001$

Cytokine analysis

For in vitro analysis, supernatants were centrifuged to pellet cellular particulates, and then flushed through a 0.22 μm syringe filter to remove remaining bacteria. For in vivo analysis, groups of three to five mice were inoculated with $1x10^4$ CFU *Y. pestis*, and sacrificed at either 24 or 48 hpi. Lungs were removed, homogenized, and centrifuged at 500 x g for 5 minutes. Lysates were treated with 100 μg/ml gentamycin and protease inhibitor for 10 min on ice before freezing at -80°C. ELISAs for IL-1β, TNFα and IFNγ were commercially obtained and were performed on total lung lysate using manufacturer protocols (BD OPTeia). ELISA sets (BD OPTeia). For Western blot analysis, lysates were run on 4–12% SDS gels (R&D Systems) and were probed with commercially available antibodies for IL-1β p17 (R&D Systems) by previously described protocols. For RT-PCR analysis, groups of three to five mice were inoculated with either $1x10^4$ *Y. pestis* or $2x10^5$ *K. pneumoniae*. At various time points, mice were sacrificed, and lungs were removed and placed in ice cold TRIzol (Gibco-BRL). Using the manufacturer protocols, RNA was purified and frozen at -80°C. cDNAs were produced using iScript reagent (BioRad) and used as templates for amplification and detection of the murine IL-1RA gene with the SYBR-Green reagent (Bio-Rad) in an iCycler thermocycler (Bio-Rad). Experiments were repeated twice to confirm trends and significance. The calculated threshold cycle (C_t) was normalized to the C_t of GAPDH gene from the same cDNA sample before calculating the fold change using the $\Delta\Delta C_t$ method. For protein analysis of IL-1RA, mice were sacrificed at 24 hpi and lungs were lavaged with 3 ml ice cold PBS. IL-1RA protein was quantified in bronchoalveolar lavage fluid using the Mouse IL-1RA/IL-1F3 Quantikine ELISA Kit (R&D Systems) per the manufacturer's instructions.

Statistics

All error was assessed as standard deviation of the mean, and significance was determined using Student's *t*-test for *in vitro* analysis (cytokine) or Mann-Whitney test for *ex vivo* analysis. Differences in survival were studied with Kaplan-Meyer analysis and the log rank test. Values of $p<0.05$ were considered significant. Analysis was performed using GraphPad Prism.

Supporting Information

S1 Fig. IL-18 cytokine activation during *Y. pestis* pulmonary infection. Mice were intranasally inoculated with fully virulent *Y. pestis* stain CO92, and sacrificed at 6, 24 and 36 hpi. Lung lysates were assessed for IL-18 cytokine protein using ELISA. N = 3 per time point.
(TIF)

S2 Fig. *In vitro* activation of IL-β by *Y. pestis* is T3SS-dependent. A. IL-1β Western blot analysis performed on supernatants obtained from bone marrow-derived macrophages (BMDMs) infected with *Y. pestis* at MOI 10. **B**. IL-1β ELISA performed on supernatants obtained from BMDMs 24 hpi with *Y. pestis* strains CO92, CO92 $pCD1^-$, CO92 Δ*lcrV*, and CO92 Δ*lcrF*, at MOI 10. All in vitro infections were performed in triplicate, with representative analysis shown.
(TIF)

S3 Fig. *In vitro* activation of IL-1β by *Y. pestis* is both NLRC4 and ASC/PYCARD- dependent. IL-1β ELISA performed on supernatants obtained from BMDMs 24 hpi with *Y. pestis* strains CO92 at MOI 10. **A**. BMDMs were derived from age-matched female wild-type, MyD88$^{-/-}$, TLR2$^{-/-}$ and TLR4$^{-/-}$ mice, and **B.** NLRP3$^{-/-}$, NLRC4$^{-/-}$, AIM2$^{-/-}$, and ASC$^{-/-}$ mice.
(TIF)

S4 Fig. IL-1 receptor is not down-regulated on the surface of phagocytic cells during *Y. pestis* infection. Wild-type and NLCR4$^{-/-}$ mice were intranasally inoculated with *Y. pestis* and sacrificed at 12 hpi. Total cells were isolated from lungs and flow cytometry was performed to detect IL-1R1 expression on cell types as previously described [4,17]. **A**. Histograms depict IL-1R1 expression in total cells isolated from mock-infected and *Y. pestis*-infected wild-type and NLRC4$^{-/-}$ mice. **B.** Mean Florescence Intensity (MFI) of IL-1R1 expression was measured for both F4/80+ macrophages and **C.** F4/80^{-} Ly6G^{+} neutrophils.
(TIF)

S5 Fig. *K. pneumoniae* and *Y. pestis* have similar replication kinetics, but differential activation of IL-1β. A. Wild-type mice were infected either with *Y. pestis* (1x10^{4} CFU) or *K. pneumoniae* (2x10^{5} CFU), and lungs were assessed for bacterial burden at 12, 24 and 36 hpi. **B**. IL-1β was detected from lung homogenates of mice at 24 hpi.
(TIF)

S6 Fig. Table of strains used for this study.
(TIF)

S7 Fig. IL-1RA protein is induced early during *Y. pestis* pulmonary infection. IL-1RA ELISA performed on bronchoalveolar lavage fluid obtained from mice 24 h after inoculation with 10^{4} CFU *Y. pestis* or PBS (mock-infected). Data represent mean ± SEM from two independent pooled experiments (n = 6-8 mice per group). * $p<0.05$ by standard t-test.
(TIF)

Acknowledgments

We thank Dr. Greg Plano for supplying the Δ1234 plasmid, Dr. Lola Stamm for supplying materials for TLR2$^{-/-}$, TLR4$^{-/-}$ and MyD88$^{-/-}$ macrophages, and Dr. Jenny Ting for materials for AIM2$^{-/-}$ macrophages. We also thank the UNC Center for Gastrointestinal Biology and Disease Histopathology Core facility (supported by NIH grant P30 DK 034987) for all histopathology services provided.

Author Contributions

Conceived and designed the experiments: VS RDP. Performed the experiments: VS RDP NMS. Analyzed the data: VS. Contributed reagents/materials/analysis tools: EAM. Wrote the paper: VS RDP WEG.

References

1. Brodsky IE, Palm NW, Sadanand S, Ryndak MB, Sutterwala FS, et al. (2010) A *Yersinia* effector protein promotes virulence by preventing inflammasome recognition of the type III secretion system. Cell Host Microbe 7: 376–387. doi: 10.1016/j.chom.2010.04.009 PMID: 20478539
2. Davis BK, Roberts RA, Huang MT, Willingham SB, Conti BJ, et al. (2011) Cutting edge: NLRC5-dependent activation of the inflammasome. Journal of immunology 186: 1333–1337. doi: 10.4049/jimmunol.1003111 PMID: 21191067
3. Latz E (2010) The inflammasomes: mechanisms of activation and function. Current opinion in immunology 22: 28–33. doi: 10.1016/j.coi.2009.12.004 PMID: 20060699

4. Miao EA, Mao DP, Yudkovsky N, Bonneau R, Lorang CG, et al. (2010) Innate immune detection of the type III secretion apparatus through the NLRC4 inflammasome. Proc Natl Acad Sci USA 107: 3076–3080. doi: 10.1073/pnas.0913087107 PMID: 20133635

5. Vladimer GI, Weng D, Paquette SW, Vanaja SK, Rathinam VA, et al. (2012) The NLRP12 inflammasome recognizes *Yersinia pestis*. Immunity 37: 96–107. doi: 10.1016/j.immuni.2012.07.006 PMID: 22840842

6. Zhao Y, Yang J, Shi J, Gong YN, Lu Q, et al. (2011) The NLRC4 inflammasome receptors for bacterial flagellin and type III secretion apparatus. Nature 477: 596–600. doi: 10.1038/nature10510 PMID: 21918512

7. Zheng Y, Lilo S, Brodsky IE, Zhang Y, Medzhitov R, et al. (2011) A *Yersinia* effector with enhanced inhibitory activity on the NF-kappaB pathway activates the NLRP3/ASC/caspase-1 inflammasome in macrophages. PLoS pathogens 7: e1002026. doi: 10.1371/journal.ppat.1002026 PMID: 21533069

8. Bubeck SS, Cantwell AM, Dube PH (2007) Delayed inflammatory response to primary pneumonic plague occurs in both outbred and inbred mice. Infection and immunity 75: 697–705. PMID: 17101642

9. Lathem WW, Crosby SD, Miller VL, Goldman WE (2005) Progression of primary pneumonic plague: a mouse model of infection, pathology, and bacterial transcriptional activity. Proc Natl Acad Sci USA 102: 17786–17791. PMID: 16306265

10. Perry RD, Fetherston JD (1997) *Yersinia pestis*—etiologic agent of plague. Clinical microbiology reviews 10: 35–66. PMID: 8993858

11. LaRock CN, Cookson BT (2012) The *Yersinia* virulence effector YopM binds caspase-1 to arrest inflammasome assembly and processing. Cell host & microbe 12: 799–805.

12. Cantwell AM, Bubeck SS, Dube PH (2010) YopH inhibits early pro-inflammatory cytokine responses during plague pneumonia. BMC immunology 11: 29. doi: 10.1186/1471-2172-11-29 PMID: 20565713

13. Bergsbaken T, Cookson BT (2007) Macrophage activation redirects yersinia-infected host cell death from apoptosis to caspase-1-dependent pyroptosis. PLoS pathogens 3: e161. PMID: 17983266

14. Lathem WW, Price PA, Miller VL, Goldman WE (2007) A plasminogen-activating protease specifically controls the development of primary pneumonic plague. Science 315: 509–513. PMID: 17255510

15. Lilo S, Zheng Y, Bliska JB (2008) Caspase-1 activation in macrophages infected with *Yersinia pestis* KIM requires the type III secretion system effector YopJ. Infect Immun 76: 3911–3923. doi: 10.1128/IAI.01695-07 PMID: 18559430

16. Cohen TS, Prince AS (2013) Activation of inflammasome signaling mediates pathology of acute P. aeruginosa pneumonia. J Clin Invest 123: 1630–1637. doi: 10.1172/JCI66142 PMID: 23478406

17. Pechous RD, Sivaraman V, Price PA, Stasulli NM, Goldman WE (2013) Early host cell targets of *Yersinia pestis* during primary pneumonic plague. PLoS pathogens 9: e1003679. doi: 10.1371/journal.ppat.1003679 PMID: 24098126

18. Sutterwala FS, Mijares LA, Li L, Ogura Y, Kazmierczak BI, et al. (2007) Immune recognition of *Pseudomonas aeruginosa* mediated by the IPAF/NLRC4 inflammasome. The Journal of experimental medicine 204: 3235–3245. PMID: 18070936

19. Miao EA, Leaf IA, Treuting PM, Mao DP, Dors M, et al. (2010) Caspase-1-induced pyroptosis is an innate immune effector mechanism against intracellular bacteria. Nature immunology 11: 1136–1142. doi: 10.1038/ni.1960 PMID: 21057511

20. Cai S, Batra S, Wakamatsu N, Pacher P, Jeyaseelan S (2012) NLRC4 inflammasome-mediated production of IL-1beta modulates mucosal immunity in the lung against gram-negative bacterial infection. Journal of immunology 188: 5623–5635. doi: 10.4049/jimmunol.1200195 PMID: 22547706

21. Mayer-Barber KD, Barber DL, Shenderov K, White SD, Wilson MS, et al. (2010) Caspase-1 independent IL-1beta production is critical for host resistance to mycobacterium tuberculosis and does not require TLR signaling in vivo. J Immunol 184: 3326–3330. doi: 10.4049/jimmunol.0904189 PMID: 20200276

22. Shenderov K, Riteau N, Yip R, Mayer-Barber KD, Oland S, et al. (2014) Cutting edge: Endoplasmic reticulum stress licenses macrophages to produce mature IL-1beta in response to TLR4 stimulation through a caspase-8- and TRIF-dependent pathway. J Immunol 192: 2029–2033. doi: 10.4049/jimmunol.1302549 PMID: 24489101

23. Montminy SW, Khan N, McGrath S, Walkowicz MJ, Sharp F, et al. (2006) Virulence factors of *Yersinia pestis* are overcome by a strong lipopolysaccharide response. Nature immunology 7: 1066–1073. PMID: 16980981

24. Zahedi KA, Uhlar CM, Rits M, Prada AE, Whitehead AS (1994) The mouse interleukin 1 receptor antagonist protein: gene structure and regulation in vitro. Cytokine 6: 1–9. PMID: 8003626

25. Dinarello CA, Novick D, Kim S, Kaplanski G (2013) Interleukin-18 and IL-18 binding protein. Front Immunol 4: 289. doi: 10.3389/fimmu.2013.00289 PMID: 24115947

26. Spinner JL, Winfree S, Starr T, Shannon JG, Nair V, et al. (2014) Yersinia pestis survival and replication within human neutrophil phagosomes and uptake of infected neutrophils by macrophages. J Leukoc Biol 95: 389–398. doi: 10.1189/jlb.1112551 PMID: 24227798
27. Jordan M, Otterness IG, Ng R, Gessner A, Rollinghoff M, et al. (1995) Neutralization of endogenous IL-6 suppresses induction of IL-1 receptor antagonist. J Immunol 154: 4081–4090. PMID: 7706746
28. Comer JE, Sturdevant DE, Carmody AB, Virtaneva K, Gardner D, et al. (2010) Transcriptomic and innate immune responses to Yersinia pestis in the lymph node during bubonic plague. Infect Immun 78: 5086–5098. doi: 10.1128/IAI.00256-10 PMID: 20876291
29. Standiford LR, Standiford TJ, Newstead MJ, Zeng X, Ballinger MN, et al. (2012) TLR4-dependent GM-CSF protects against lung injury in Gram-negative bacterial pneumonia. American journal of physiology Lung cellular and molecular physiology 302: L447–454. doi: 10.1152/ajplung.00415.2010 PMID: 22160309
30. Willingham SB, Allen IC, Bergstralh DT, Brickey WJ, Huang MT, et al. (2009) NLRP3 (NALP3, Cryopyrin) facilitates in vivo caspase-1 activation, necrosis, and HMGB1 release via inflammasome-dependent and-independent pathways. Journal of immunology 183: 2008–2015.
31. Kim S, Bauernfeind F, Ablasser A, Hartmann G, Fitzgerald KA, et al. (2010) Listeria monocytogenes is sensed by the NLRP3 and AIM2 inflammasome. European journal of immunology 40: 1545–1551. doi: 10.1002/eji.201040425 PMID: 20333626
32. Jessen DL, Osei-Owusu P, Toosky M, Roughead W, Bradley DS, et al. (2014) Type III secretion needle proteins induce cell signaling and cytokine secretion via Toll-like receptors. Infect Immun 82: 2300–2309. doi: 10.1128/IAI.01705-14 PMID: 24643544
33. Holgate ST (2012) Innate and adaptive immune responses in asthma. Nature medicine 18: 673–683. doi: 10.1038/nm.2731 PMID: 22561831
34. Mao XQ, Kawai M, Yamashita T, Enomoto T, Dake Y, et al. (2000) Imbalance production between interleukin-1beta (IL-1beta) and IL-1 receptor antagonist (IL-1Ra) in bronchial asthma. Biochemical and biophysical research communications 276: 607–612. PMID: 11027520
35. Frank JA, Pittet JF, Wray C, Matthay MA (2008) Protection from experimental ventilator-induced acute lung injury by IL-1 receptor blockade. Thorax 63: 147–153. PMID: 17901159
